# Chiari III Malformation on Prenatal and Postnatal Imaging Complicated by Syndrome of Inappropriate Secretion of Anti-diuretic Hormone (SIADH) and Serratia marcescens Meningitis

**DOI:** 10.7759/cureus.17327

**Published:** 2021-08-20

**Authors:** Ryan McGeary, Chetan Shah

**Affiliations:** 1 Radiology, Western University of Health Sciences, Pomona, USA; 2 Pediatric Radiology, Nemours Children's Health System, Jacksonville, USA

**Keywords:** chiari iii malformation, serratia marcescens, fetal mri, occipital encephalocele, pediatric hydrocephalus, gram-negative meningitis, siadh

## Abstract

Among various types of Chiari malformations (CMs), CM III is the most infrequently encountered. In this article, we present a case of CM III with occipital cephalocele appreciated on both prenatal imaging and postnatal follow-up MRI. This case illustrates not only the evolution of this malformation from the in-utero images of fetal MRI to the newborn MRI but also highlights the complications that may accompany this diagnosis such as hydrocephalus and infection. The patient also developed syndrome of inappropriate secretion of anti-diuretic hormone (SIADH). The most current thoughts on the pathophysiology of this entity are also reviewed along with an approach to the differential diagnosis and treatment.

## Introduction

Hans Chiari described a group of congenital hindbrain malformations in 1891, which are named after him as various Chiari malformations. Originally, he described three types: Chiari malformations I, II, and III. A few years later, he added the fourth type: Chiari IV malformation. Recently, a new classification is being used that includes the Chiari 0, Chiari 1.5, and Chiari 3.5 malformations [1-2]. Chiari 3.5 malformation is incompatible with life. A Chiari III malformation is a rare entity, comprising only a small percentage of all various types of Chiari malformation.

We present prenatal and postnatal MRI of a case of Chiari III malformation that showed the progression of the abnormality with cerebellar tissue herniating in the defect on postnatal MRI but not on prenatal MRI. Syndrome of inappropriate secretion of anti-diuretic hormone (SIADH) associated with intracranial diseases and neurosurgical procedures like posterior fossa decompression have been described. However, to our knowledge, SIADH in the setting of Chiari III malformation has not been described. Also, the infant developed shunt catheter-related infection after four weeks of shunt placement causing Serratia marcescens meningitis and subdural empyema that was treated with the evacuation of empyema, subdural drain, intravenous and intrathecal antibiotics. We demonstrate the cephalocele resolving over multiple MRIs performed over the first 14 months of life without any focal surgical intervention at the occipital defect.

Prognosis in patients with occipital cephaloceles depends on the presence or absence of the following: neural tissue within the cephalocele, hydrocephalus, and infection. In addition, the size of the cephalocele and associated anomalies determine the overall prognosis [3].

## Case presentation

**Figure 1:**
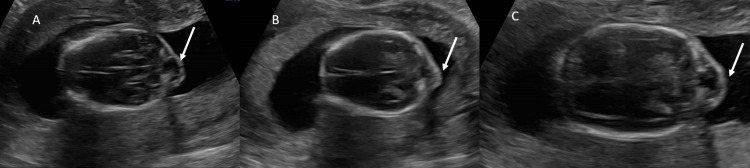
Prenatal Ultrasound: Chiari III Malformation Ultrasound axial image of the brain (A-C) performed at 17 weeks six days of gestation shows occipital cephalocele (arrows)

**Figure 2:**
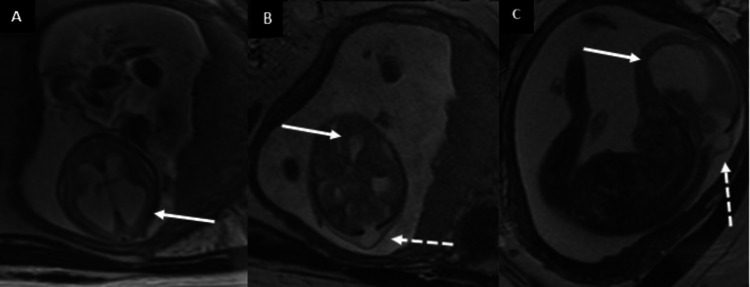
Fetal MRI: Chiari III Malformation Axial (A, B) and sagittal (C) T2-weighted sequences (A-C) demonstrate marked dilatation of the lateral ventricles (solid arrows). In images B and C, there is a cerebrospinal fluid (CSF) intensity outpouching through a midline occipital bone defect (dashed arrows).

A 39-year-old pregnant woman (gravida 2, para 1) presented to an outpatient obstetrics clinic for prenatal care. Ultrasound performed at 17 weeks six days of gestation demonstrated ventriculomegaly and occipital encephalocele (Figure 1). Her prenatal course was significant for pre-gestational diabetes, though well-controlled, during her pregnancy with an average hemoglobin A1c of 6.5%. Her screening beta-human chorionic gonadotropin (beta-HCG) and PAPP-A were negative. An MRI was obtained at 21 weeks of gestation, which confirmed the presence of a midline defect in the occipital bone with an associated occipital cephalocele, noted in Figure 2. Cephalocele contained cerebrospinal fluid (CSF) and meninges. No brain tissue was seen herniating into the cephalocele. The overlying skin was intact. Additionally, the lateral ventricles were markedly enlarged. The septum pellucidum was absent and the falx cerebri was present.

**Figure 3:**
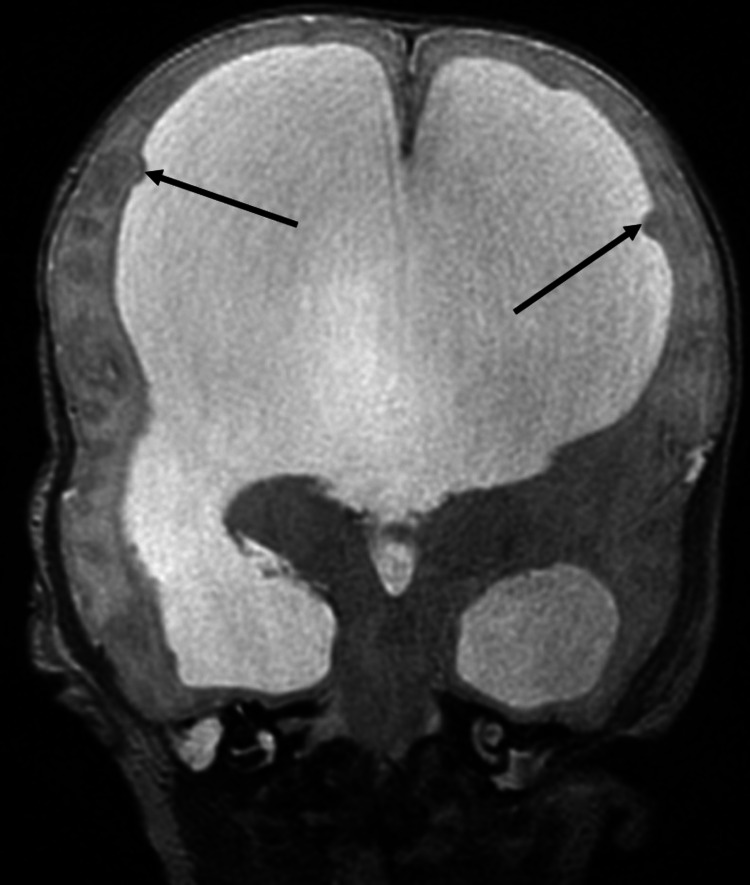
Neonatal MRI: Heterotopia Coronal T2-weighted MRI image demonstrates severe dilatation of the lateral ventricles. Multiple gray matter heterotopias (arrows) that are isointense to gray matter are seen along the lining of lateral ventricles.

**Figure 4:**
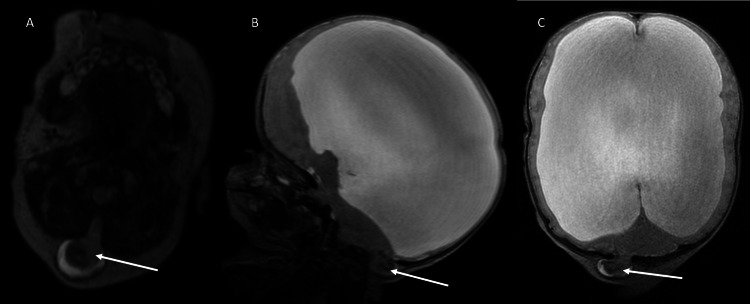
Occipital Encephalocele Axial (A), sagittal (B), and coronal (C) T2-weighted images of the brain MRI performed on Day 1 of life show cerebellar tissue (arrow) extending into the occipital cephalocele. Dilated lateral ventricles are also seen.

The fetus was delivered at 39 weeks of gestation via cesarean section without immediate complication. On Day 1 of life, an MRI brain exam was performed, demonstrating severely dilated lateral ventricles along with scattered subependymal grey matter heterotopia as shown in Figure 3. The midline occipital bone defect was again identified although on this exam was interval herniation of cerebellar parenchyma (Figure 4) in the cephalocele, which also contained CSF and meninges.

Later, on Day 1, a ventriculoperitoneal shunt (VP shunt) was inserted on the right side. MRI performed on Day 6 of life showed a decrease in the size of the lateral ventricles and the development of bilateral subdural fluid. The cerebellar tissue was no longer seen herniating within the cephalocele. The size of the cephalocele remained stable and contained CSF and meninges. The patient was discharged from the hospital on the thirteenth day of life.

**Figure 5:**
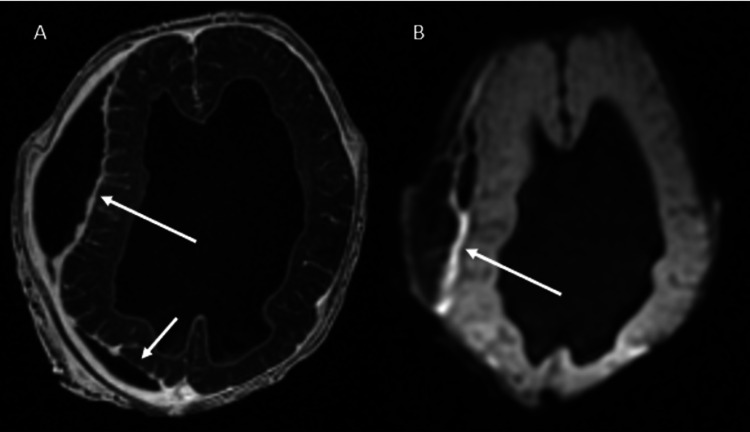
Subdural Empyema at Seven Weeks Axial post-contrast MRI of the brain (A) shows right-sided subdural fluid with peripheral enhancement (arrow). Axial diffusion-weighted MRI (B) shows restricted diffusion (arrow) within the right-sided subdural fluid consistent with subdural empyema.

Unfortunately on Day 41 of life, the infant presented in the emergency room with fever and fussiness. The VP shunt was tapped and CSF sent for culture. CSF culture showed infection with the gram-negative bacteria Serratia marcescens. The patient was admitted to the pediatric intensive care unit (PICU) and an intravenous antibiotic (Meropenem) was given. The patient also received 10 days of intrathecal gentamicin. On Day 44, the right VP shunt was removed and a right external ventricular drain (EVD) was placed. The patient developed right subdural empyema (Figure 5), which was evacuated and a subdural drain was placed on Day 50 of life. On Day 76 of life, the subdural drain and EVD were removed and a VP shunt was placed on the left side. During the six-week stay in the pediatric intensive care unit (PICU), the infant experienced hyponatremia that was treated initially by oral sodium chloride supplementation. Blood work revealed low serum osmolarity, normal blood urea nitrogen, low serum creatinine, and the diagnosis of syndrome of inappropriate secretion of anti-diuretic hormone (SIADH) was established. Sodium chloride supplement was stopped and restriction of fluid to 400 ml per day was initiated. The sodium level returned to normal levels. The patient was discharged on Day 82 of life.

The remaining postnatal course was complicated by infantile spasms and seizures that have been controlled by oral levetiracetam and lacosamide with few breakthrough seizures.

**Figure 6:**
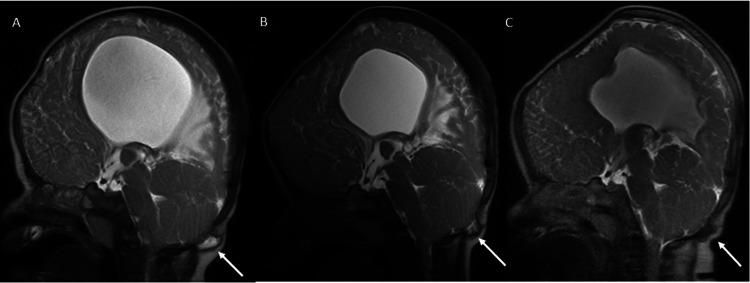
Resolution of the Occipital Cephalocele Sagittal single-shot fast spin-echo (SSFSE) images of the brain MRI performed at eight months (A), 12 months (B), and 14 months (C) show a gradual decrease in the size of the occipital cephalocele (arrows).

Multiple MRIs (Figure 6) performed over the first 14 months of life show gradual resolution of the cephalocele without any focal surgical intervention at the occipital defect.

## Discussion

Currently, there are six different Chiari malformations (CM) that have been described. Chiari I malformation (CM I) is the most commonly encountered, defined by the inferior descent of the cerebellar tonsils below the plane of the foramen magnum by 6 mm or more [1]. Once the brainstem becomes involved in this descent, as seen by inferior displacement of the obex, the designation Chiari malformation 1.5 (CM 1.5) can be used. Both these may be associated with syrinx formation. The presence of syrinx alone without tonsillar herniation or another secondary cause defines Chiari malformation (CM 0). Both CM 0 and CM 1.5 are considered variants of CM I [1]. The designation of CM II is used for the descent of the cerebellar tonsils and brainstem below the foramen magnum that is almost invariably associated with lumbar myelomeningocele, giving distinct features in the brain such as superior displacement of the cerebellum, tectal beaking, small posterior fossa, and enlarged massa intermedia. As described in this case, CM III involves herniation of meninges with or without posterior fossa contents through a defect in the occiput or C1/C2. Few cases have been described in the literature regarding a variant of CM III, called CM 3.5, described by Hans Chiari himself in 1891 [2]. This variant also involves herniation of cerebellar/brainstem tissue through an occipital defect but with clear communication with the stomach [2]. The last Chiari malformation type is the Chiari IV malformation (CM IV), often erroneously thought of as isolated cerebellar hypoplasia. In keeping with Hans Chiari’s original account of this entity, true CM IV is detailed as an occipital encephalocele that contains supratentorial tissue with concomitant cerebellar hypoplasia [4].

Chiari malformation type III is by far the most rarely seen of all the described Chiari malformations, representing approximately 1%-4.5% of these deformities [4-6]. The etiology of Chiari malformation is thought to result from an aberrancy in the process of dorsal induction whereby the embryonic neural tube is formed and closed [5-8]. This process begins at the future craniocervical junction and subsequently extends craniocaudal at approximately 3-5 weeks of gestation [5-6]. The most current theory postulated by McLone and Knepper states that due to defective molecular signaling, the newly formed neural tube fails to close. As a result, the open neural tube allows cerebrospinal fluid (CSF) to leak into the surrounding intrauterine milieu [6-7]. Without CSF in the central canal of the cord, distention of the ventricular system does not occur, which is thought to otherwise induce neural and calvarial development [6-7]. Subsequently, the malformed posterior fossa becomes vulnerable to herniation through any concomitant calvarial defect. A definitive cause for this malformation has yet to be elucidated though, in general, folate deficiency has been shown to increase the risk of neural tube defects such as Chiari II and III [9]. However, in Chiari III malformation, the CSF does not leak into the amniotic fluid due to intact skin overlying the cephalocele.

Clinically, CM are often diagnosed during the antenatal course by fetal ultrasound or fetal MRI. Little data are available regarding the postnatal course of this deformity though these patients typically have a high mortality rate. For patients surviving past the neonatal period, there is often severe neurologic sequela such as epilepsy, spasticity, and mental retardation.

Although CM II and CM III are distinct entities, brain MRI may show similar features as in CM II in addition to occipital meningocele/encephalocele. The major difference is that in CM II, there is no skin covering the meningocele, whereas, in CM III, there is skin covering the meningocele/encephalocele. Hence, the nervous tissue is not in direct contact with the amniotic fluid and there are no biomarkers in CM III unlike that in CM II.

Without available biomarkers, the diagnosis of CM III is based primarily on imaging. Suspicions for this entity are often first brought about on obstetric ultrasound where a small posterior fossa is seen with a meningeal outpouching at the occipital or high cervical region. Follow-up MRI is the standard for anatomic diagnosis and will confirm the presence of a variable-sized CSF intensity meningocele with or without cerebellar/brainstem tissue within the defect. This herniation occurs through an osseous defect, either in the occipital bone or through a dysraphism at C1 or C2. If herniated cerebellar/brainstem tissue is present, MRI can also characterize if the tissue is hemorrhagic, causing low signal on T2/T2* weighted sequences or gliotic causing high signal.

MRI in CM III may identify concomitant abnormalities usually seen in Chiari II malformation (CM II) such as callosal dysgenesis, polymicrogyria/stenogyria, and tectal beaking, which may or may not be present. Hydrocephalus is an additional feature to be aware of, present in 22% of cases [5]. MR venography (MRV) can also be useful for the preoperative delineation of the venous sinus anatomy surrounding the meningocele/myelomeningocele, as duplication of the superior sagittal sinus has been reported in these patients [10-13]. Additionally, dural sinuses may herniate along with cerebellar/brainstem tissue, which can be seen in up to 50% of cases [14].

Occipital or high cervical osseous defects can also be well-delineated on CT in addition to other osseous abnormalities such as clival scalloping or skull defects known as “lacunar skull.” Lacunar skull in particular manifests as a beaten-silver appearance or lückenschädel, a German word (lücke means hole and schädel means skull), of the skull due to membranous bone dysplasia, present in up to 80% CM II cases but present to a variable degree in CM III [8]. If this feature is present, it will often resolve by six months and is not associated with hydrocephalus [5]. The use of MR angiography may also provide benefit, particularly when there is brainstem herniation that may stretch and kink the basilar artery.

The differential diagnosis (Table 1) regarding CM III is relatively straightforward and relies on the presence of secondary findings that are often found with other entities. One diagnosis to consider is an isolated occipital encephalocele. These cases can be differentiated from CM III by the fact that they do not involve the foramen magnum and lack any findings seen with CM II, which often are present in CM III [15]. There are also various syndromic causes of an occipital encephalocele such as Meckel Gruber syndrome, Mullerian duct, and renal agenesis, cervicothoracic somite dysplasia (MURCS), and Walker-Warburg syndrome. These syndromes will demonstrate additional secondary findings such as post-axial polydactyly in Meckel-Gruber syndrome or “kinking” at the pontomesencephalic junction in Walker-Warburg syndrome [16].

**Table 1:** Differential diagnosis

	Chiari III Malformation	Isolated Occipital Encephalocele	Other Syndromic Encephaloceles
US	Herniation meninges with or without cerebellar tissue. + or - Hydrocephalus	Herniation meninges with or without cerebellar tissue	Polydactyly, Dandy-Walker continuum, microtia, and cardiac/GU anomalies
CT	Osseous defect at occiput or C1/2	Osseous defect spares the foramen magnum	Occipital bone defect + or – partial/complete absence of cervicothoracic vertebrae
MRI	May have secondary brain anomalies found with CM II such as callosal dysgenesis, tectal beaking, and schizencephaly.	No associated brain anomalies	Look for concomitant abnormalities found in syndromic causes such as polydactyly, pontomesencephalic kinking, and cardiac/GU anomalies

Treatment of CM III involves surgical repair of the herniation sac and cerebrospinal fluid diversion if accompanied by hydrocephalus. If CSF diversion is needed, better outcomes are found when the diversion takes place before the encephalocele repair [17]. Resection of an encephalocele may not be of any benefit if parenchymal contents of the hernia sac are greater than that of the cranial vault. Factors that portend a worse prognosis are the presence of hydrocephalus and infection in addition to having a large hernia sac and increased neural contents within the herniation [18].

Hyponatremia in neurosurgical patients has been described as resulting from either SIADH or cerebral salt wasting (CSW) [18]. CSW requires vigorous salt and volume replacement. In contrast, SIADH requires fluid restriction. Hence, differentiation between these two entities is important. Our case had hyponatremia related to SIADH and fluid restriction helped resolve hyponatremia.

## Conclusions

Chiari III malformation is a rare entity, comprising only a small percentage of Chiari malformations. This case illustrates both the fetal MRI and neonatal MRI appearance of this deformity and highlights the potential complications that may arise such as hydrocephalus and infection. Both these complications foreshadow a worse prognosis and should be addressed before surgical repair of the underlying cephalocele. SIADH that developed in the setting of Chiari III malformation that required multiple neurosurgical procedures resolved with fluid restriction.
